# Is there a bidirectional interaction between periodontitis and the severity of SARS-CoV-2 infection?

**DOI:** 10.17179/excli2021-3810

**Published:** 2021-06-01

**Authors:** Rayle Monteiro Andrade, Raquel Souza Marques, Tatiana Rodrigues de Moura, Saul Martins de Paiva, Ricardo Queiroz Gurgel, Paulo Ricardo Martins-Filho

**Affiliations:** 1 Graduate Program in Dentistry, Federal University of Sergipe, Aracaju, Sergipe, Brazil; 2 Investigative Pathology Laboratory, Federal University of Sergipe, Aracaju, Sergipe, Brazil; 3 Graduate Program in Health Sciences, Federal University of Sergipe, Aracaju, Sergipe, Brazil; 4 Graduate Program in Dentistry, Federal University of Minas Gerais, Belo Horizonte, Minas Gerais, Brazil; 5 Department of Medicine, Federal University of Sergipe, Aracaju, Sergipe, Brazil

## ⁯

***Dear Editor,***

Several diseases, such as diabetes, obesity, vascular diseases, and lung diseases including pneumonia and chronic obstructive pulmonary disease (COPD) have been strongly associated with periodontitis, even though the mechanisms or causal associations have not been completely established. Periodontitis is one of the most common diseases of the oral cavity and is characterized by the progressive destruction of the tissues supporting the tooth (Listgarten, 1986[3]). The interaction between subgingival polymicrobial biofilm and the host cells leads to the release of pro-inflammatory cytokines, especially interleukin-1 (IL-1), IL-6 and tumor necrosis factor (TNF) with important clinical outcomes on periodontal tissues and repercussion to the systemic inflammatory burden (Pan et al., 2019[5]). 

Severe acute respiratory syndrome coronavirus 2 (SARS-CoV-2) is a novel single-stranded RNA-enveloped virus primarily transmitted person-to-person by close contact through respiratory droplets and is the causal agent of the coronavirus disease 2019 (COVID-19). SARS-CoV-2 can infect cells with angiotensin-converting enzyme 2 (ACE2) receptor leading to a wide range of symptoms including fever, respiratory manifestations, gastrointestinal disorders, and cardiac and renal injury. Recent studies have shown the presence of SARS-CoV-2 in periodontal tissues, even many days after the first symptoms (Fernandes Matuck et al., 2020[1]). This study performed a post-mortem biopsy in seven patients and the periodontal tissue was positive for SARS-CoV-2 in five of them, which may suggest that this tissue may be a repository for the virus. 

For some patients with COVID-19, the binding of the SARS-CoV-2 spike protein to the ACE2 leads to a massive immune response with an increased release of cytokines, especially IL-6 implicated in multi-organ damage and risk of death (Qin et al., 2020[7]). This dysregulated inflammatory response also occurs in periodontal disease and can cause over-stimulation of the immune system (Lamont et al., 2018[2]), which could aggravate the severity of SARS-CoV-2 infection due to the cytokine storm. Otherwise, we also hypothesized a bilateral association. In severe cases of COVID-19, patients are hospitalized and need mechanical ventilation, the length of stay in hospital environment is prolonged, requiring different types of medication and, in many cases, oral hygiene is neglected (Pitones-Rubio et al., 2020[6]). Consequently, persistent alterations in the oral microbiome and the increased burden of pro-inflammatory cytokines can impose detrimental effects on the periodontal disease. 

A recent case-control study of 568 patients with COVID-19 showed that patients with periodontal disease were 3.5 times more likely to be admitted to an intensive care unit, 4.5 times more likely to need mechanical ventilation and had 8.8 times more chance of death compared to those with good periodontal health (Marouf et al., 2021[4]). Serum markers of systemic inflammation were significantly higher in patients with COVID-19 who had periodontitis, suggesting a synergic interaction between infections leading to increased rates of adverse outcomes in COVID‐19 patients. 

There is growing evidence to suggest that inflammation resulting from periodontitis may be a risk factor for the severity of COVID 19. Further studies relating periodontitis and patients with COVID-19, including mild and severe forms of the disease, should investigate this association. Strengthening this evidence may allow to identify novel prognostic indicators for patients with COVID-19 and to offer a preventive approach regarding oral care and hygiene.

## Funding

There is no funding source. 

## Conflicts of interest

There is no conflict of interest.
